# Assessment of selected molecular factors and 17-β estradiol dosage in response to *Toxoplasma gondii* infection in swine

**DOI:** 10.14202/vetworld.2022.1641-1649

**Published:** 2022-07-13

**Authors:** Annamaria Castello, Esterina Fazio, Tiziana Alfonzetti, Renato Paolo Giunta, Antonio Salvaggio, Alida Maria Ferlazzo, Cristina Cravana, Giuseppe Bruschetta, Pietro Medica, Anna Maria Fausta Marino

**Affiliations:** 1 Department of Catania, Istituto Zooprofilattico Sperimentale Della Sicilia, Catania, Italy; 2 Department of Veterinary Sciences, University of Messina, Polo Universitario dell’Annunziata, Messina, Italy

**Keywords:** biomarkers, host-parasite interaction, swine, *Toxoplasma gondii*

## Abstract

**Background and Aim:**

*Toxoplasma gondii* is a global zoonotic parasite infecting virtually all warm-blooded species, although a species-specific variability is evident referring to symptoms frame. Both the success of *T. gondii* and the outcome of infection depend on a delicate balance between host cellular pathways and the evasion or modulation strategies elicited by the parasite. The hormonal and molecular mechanisms involved in this delicate host-parasite balance are still unclear, especially when considering intermediate host species other than mouse. This study aimed to assess any correlation between *T. gondii* infection and selected molecular and hormonal factors involved in responses to infection in susceptible species such as swine. Moreover, blood counts and hematochemical assays (glucose, total cholesterol, and triglycerides dosage) were performed to evaluate the overall health condition of animals.

**Materials and Methods:**

Toxoplasmosis was diagnosed by enzyme-linked immunosorbent assay for antibodies determination and real-time polymerase chain reaction (RT-PCR) for *T. gondii* DNA detection. Target genes coding for key factors of cell responses to *T. gondii* infection were selected, and their transcription was assessed in various tissues by quantitative RT-PCR. 17-β estradiol concentrations were assessed by *fluorimetric enzyme-linked immunoassay* and the AIA-360 automated immunoassay analyzer. Blood count and hematochemical analyses were performed by a blood cell counter and a spectrophotometer, respectively.

**Results:**

The present research highlighted significant differences among infected and uninfected swine (control group) for both transcription profiles of some of the molecular factors considered and 17-β estradiol concentrations. Referring to the assessed hematological and biochemical parameters, no statistically significant differences were observed in infected swine compared to the control group.

**Conclusion:**

Our results contribute to the enrichment of data available about the subject and could be useful for a deeper knowledge of the interaction between this parasite and its hosts. However, more aspects are still unclear, such as the effective response of downstream molecules from the same pathways to the variation of factors observed in this study either assessing how the same factors respond to *Toxoplasma gondii* infection in other host speciesand further analyses should be performed on other host species.

## Introduction

*Toxoplasma gondii* is an intracellular parasite spread worldwide that infects virtually all warm-blooded animals, including humans. Approximately one-third of the human population is infected by this zoonosis, even though in most people, symptoms (when present) are mild and mimic other diseases [1]. Nevertheless, toxoplasmosis can lead to rather ominous outcomes, including death, especially in immunocompromised individuals and neonates. This infection is usually subclinical among animals but can be fatal in some hosts [2]. In fact, no clinical signs are observed in infected poultry and rare febrile and ocular symptoms occur in equids and cattle. However, severe symptoms are more common in swine (ocular and nasal discharge, dyspnea, cyanosis, neurological signs, occasional reproductive failure, abortion, and death), sheep, and goats (frequent abortions) [3]. Even though hosts clear the majority of parasites during acute infection, surviving parasites persist as slow-growing bradyzoite tissue cysts. These are most abundant in tissues such as brain, eye, cardiac, and skeletal muscle [4]. Parasite-dependent factors can affect the outcome of infection, as suggested by an increasing number of references reporting the isolation of non-archetypal genotypes of *T. gondii* from hosts affected by severe symptoms. These observations refer to humans and animals, although the atypical genotypes associated with poorer symptoms differ between humans [5, 6] and animals [7, 8].

Rather numerous epidemiological data support the association between host species and susceptibility. Furthermore, some authors report or suggest a correlation between symptom severity and host-dependent factors such as age and gender. Still, the molecular and hormonal mechanisms underlying host response to *T. gondii* infection are not exhaustively defined yet, especially when considering that most of the studies are performed on the murine model following experimental infection. This impedes any evaluation on other hosts and interspecies comparisons.

This study aimed to carry out a preliminary assessment of correlations between *T. gondii* infection and molecular factors of swine, as an alternative example of the intermediate host, particularly susceptible to *T. gondii*.

## Materials and Methods

Expression profiles were evaluated on selected molecules for which either the previous study [9] has already suggested or highlighted a role in the delicate host-parasite balance or because they constitute the key factors of cellular mechanisms that can be influenced by *T. gondii*. In addition, the analyses were performed on different animal tissues to highlight any differences between them. Since involvement of sex hormones in host response to infections was suggested [9], 17-β estradiol (E2) concentration in females was also measured to evaluate its correlation to *T. gondii* infection in swine. Blood counts and hematochemical assays (glucose, total cholesterol, and triglycerides) were performed to evaluate the overall health condition of animals and to reveal any interplay between physiological parameters and transcription of the assessed molecular factors.

### Ethical approval

All analyses were performed on animals to be slaughtered for human consumption. The latter was carried out in compliance with the Italian and European reference legislation, which guarantees animal protection at slaughter. The Committee for the Care and Use of Animals of Messina University (Italy) concluded that the proposed study did not need ethical approval since, according to Italian law, it was not considered an animal experiment.

### Study period and location

The investigations were conducted on swine used for meat production, reared in the Sicilian territory, and subjected to slaughter from June to August 2020 in a slaughterhouse attached to a farm in the province of Enna, Sicily, Italy.

### Animals

The analyses described below were performed on 38 swine randomly selected from 10 different farms, among which 19 females and 19 males. Experimentally infected animals were not included in this study and the ones that tested positive for toxoplasmosis were naturally infected. Animals were given progressive numbers from 1 to 38 based on the sampling order at the time of slaughter. None of the sampled animals showed clinical signs at the time of slaughter. Samples collected and the corresponding analyses performed are listed in Table-1.

**Table 1:** Tissues sampled and corresponding performed analyses.

Samples collected	Analyses performed
Blood	Serological diagnosis of Toxoplasmosis (ELISA) Blood count Hematochemical parameters (Glucose, total cholesterol and triglycerides) Dosage of 17-β estradiol
Brain tissue, eye bulb, cardiac muscle, skeletal muscle and caecum	Molecular assessment of *T. gondii* tissues contamination
Brain tissue, skeletal muscle, caecum and lymph node	Gene expression profile (transcription)

Among listed samples, the tissue sections to be processed for the molecular diagnosis of *T. gondii* contamination were put in sterile containers, while small aliquots of brain tissue, skeletal muscle, cecum, and lymph node, intended for gene expression evaluation, were immediately placed in sterile RNase-free containers containing RNAlater solution (Applied Biosystems, Waltham, Massachusetts, USA), to preserve them from RNase degradation. All samples were maintained refrigerated until processing.

### Serological diagnosis of toxoplasmosis

Blood samples (15 mL) from each swine was collected from a jugular vein at the time of slaughter, and put in a tube for serum isolation (evacuated tubes; Z serum clot activator, Vacuette®, Greiner Bio-One, Kremsmünster, Austria), and immediately refrigerated at 4°C. Diagnosis of infection was performed by an ACCREDIA accredited *enzyme-linked immunosorbent assay* (ELISA) method using thePriocheck Ab porcine kit (Applied Biosystems) [10]. Optical densities were measured at l = 450 nm, using the microplate reader BIO-RAD 680, BIO-RAD Laboratories, Segrate, Italy. Sample positivity percentages (SP%) were calculated and processed according to the calculation formula and discrimination criteria indicated by manufacturers.

### Molecular assessment of *T. gondii* tissues contamination

To assess whether any of the most common *T. gondii* target tissues or organs were contaminated by infectious forms of the parasite, various sections were collected postmortem from each animal, transferred into sterile stomacher bags, and blended with a mortar. The homogenate was diluted with an equal volume of tris-ethylenediaminetetraacetic acid buffer and rehomogenized with a stomacher. DNA extraction was performed on a volume of 200 μL of homogenate using the nexttec™ 1-Step DNA Isolation Kit for Tissues and Cells (Nexttec Biotechnologie GmbH, San Lazzaro di Savena, Italy), according to the manufacturer’s instructions. DNA was quantified by NanoDrop® ND-1000 spectrophotometer (Thermo Fisher Scientific, Waltham, Massachusetts, USA). An ACCREDIA accredited real-time polymerase chain reaction (RT-PCR) method targeting the 529 bp repeated fragment of *T. gondii* genome (GenBank accession no. AF146527) was applied to sampled tissues [11]. Each batch of PCR assays included *T. gondii* genomic DNA (ATCC 50174D™ from Rh-88 *T. gondii*; LGC Standards) as a positive control template and molecular grade water as a negative control.

### Blood count and hematochemical analyses

Blood count was performed by the eCoVet counter, SEAC (SEAC, Florence, Italy). Hematological parameters assessed are listed below: Hematocrit, hemoglobin, red blood cell, white blood cell, neutrophils, lymphocytes, and platelets. Hematochemical analyses were performed using spectrophotometric kits (Spinreact, Girona, Spain) by GOD/POD/PAP, CHOD/POD/PAP, and GPO/POD/PAP method for glucose, total cholesterol, and triglycerides dosage, respectively.

### Dosage of 17-β estradiol

17-β estradiol concentrations were assessed twice for each animal (19 female piglets of 1 year) by fluorimetric enzyme-linked immunoassay and the AIA-360 automated immunoassay analyzer (TosohBioscience, Belgium-Japan). The intra- and inter-assay variation coefficients for 17-β estradiol concentrations were 3.8% and 9.1%, respectively.

### Target genes selected for the analyses

A series of molecules involved in different cellular mechanisms were selected for which several studies, carried out mostly *in vitro*, showed involvement in cellular responses to *T. gondii*. These molecules are listed below.

- Hypoxia-inducible factor 1 (HIF1) is a heterodimer consisting of α and β subunits. It regulates various pro-parasite genes, such as glycolytic metabolic genes, transferrin receptors, and vascular endothelial growth factors, and plays a fundamental role in the growth and survival of the parasite inside the host cell. *In vitro* experiments demonstrated that *T. gondii* activates HIF by both stimulating HIF1α subunit transcription [12] and stabilizing this protein [13].
- Prolyl hydroxylases (PHDs) are HIF inhibitors that hydroxylate-specific prolyl residues of the HIFα subunit, resulting in its proteolysis. Although three HIF PHD isoforms were identified that have the potential to catalyze this reaction, namely, PHD1, PHD2, and PHD3, each of them contributes in a non-redundant manner to the regulation of HIF-α subunits. In particular, PHD2 has a dominant role in a wide range of cell types, while PHD1 and PHD3 are mostly expressed in testicles and heart cells, respectively.
- AKTs are serine-threonine kinases involved in several cellular functions (such as proliferation, migration, apoptosis, and angiogenesis) and metabolic pathways. In fact, they act downstream of the insulin-like growth factor 1/phosphatidylinositol 3-kinase (PI3K)-mediated response to insulin. In mammals, this protein family includes three isoforms, namely, AKT1 protein kinase B α (PKBα), AKT2 (PKBβ), and AKT3 (PKBɣ), which differ from each other in their tissue localization. Moreover, they have some shared functions and some isoform-specific ones. In particular, AKT1 plays a key role in apoptosis and cell proliferation processes, mediating the transition from the G1 to the S phase of the cell cycle. Together with AKT1, also PI3K and mitogen-activated protein kinase 3 (MEK3) play a key role in processes such as apoptosis and cell proliferation.
- Nucleotide-binding and oligomerization domain (NOD) (NOD-like receptor) are cytoplasmic receptors that are part of the pattern recognition receptors, together with other receptors, such as toll-like receptors. They can recognize structures associated with pathogenic microorganisms (pathogen-associated molecular patterns) or with cell damage (damage-associated molecular patterns [DAMPs]), activating the innate immune response. Furthermore, these receptors can trigger a type of inflammatory cell death known as pyroptosis. Nod2 is a cellular receptor capable of recognizing *T. gondii* and mediating the host’s innate immune responses; therefore, it plays a fundamental role in host control of parasite replication.
- The guanosine triphosphatases (GTPases) family includes various proteins involved in interferon (IFN)-mediated immune responses. The mRNA relative quantitation was carried out for the immunity-related GTPase C (IRGC), guanylate-binding protein (GBP)1, GBP5, and very large inducible GTPase (VLIG) coding genes (IFNɣ-inducible IRGC, GBP 1, GBP 5, and VLIG, respectively). Especially GBPs and IRGs play a crucial role in restricting pathogen growth early after infection. In particular, GBP1 targets macrophages with various pathogen-containing vacuoles, including *T. gondii*, and promotes both pyroptosis and apoptosis [14], while IRGs attack *T. gondii-*infected cells and trigger necrotic death, killing at least some of the parasites. Furthermore, experiments performed in mice demonstrated that in dying the host cells release a protein that can stimulate local immunity. For most strains of *T. gondii*, this early immune attack is probably enough to prevent the parasite from killing the host and allows the parasite to establish a long-lasting infection in tissues such as brain or muscle [15].

### Quantitative RT-PCR

Total RNA was extracted by RNeasy mini kit (Qiagen, Hilden, Germany) following the manufacturer’s instructions, then subjected to gDNA removal and conversion to cDNA using the QuantiNova Reverse Transcription Kit (Qiagen). Quantitative PCR was performed on the target genes described above, using SYBR green probes (PowerUp SYBR Green Master Mix, Applied Biosystems) and the 7300 RT-PCR thermal cycler (Applied Biosystems). Threshold cycle (Ct) values detected for the selected target genes were normalized to the housekeeping gene *β-actin*. For each target gene, the calibrator was chosen, that is, the analyzed tissue in which the target gene is the least expressed and expression relative to the calibrator was calculated using the ΔΔCt method and the Microsoft Excel software. Data were analyzed using unpaired two-tailed Student’s t-test. p < 0.05, 0.01, or 0.001 was considered to indicate statistical significance.

## Results

Among the 38 pigs sampled, 19 tested positive for *T. gondii* infection and 19 were uninfected, as they tested negative for both indirect (antibodies detection in sera by ELISA assay) and direct (detection of *T. gondii* DNA in tissues by RT-PCR) diagnostic examinations. Infected animals were found in seven out of 10 farms subjected to sampling. In particular, as shown in Table-2, 11 out of 19 infected animals tested positive for both indirect and direct diagnostic examinations, while three and five out of 19 infected animals tested positive only for the indirect and only for the direct diagnostic assessment, respectively.

**Table 2:** Results of diagnostic examinations.

Sampled animals			Infected				Uninfected
	
Total	Males	Females	Total	Serum/tissues	Serum	Tissues	
38	19	19	19	11	3	5	19

The majority of infected animals were reported as contaminated at skeletal muscle, while for none of them, *T. gondii* DNA was detected in cecum. Diagnostic results obtained for infected swine are detailed in Table-3.

**Table 3:** Diagnostic results obtained for infected animals.

Animal ID	Serological test result		Analyzed tissues where *T. gondii* DNA was detected

	Positive	Negative	
1		•	Skeletal muscle
3		•	Skeletal muscle, eye bulb
4	•		Skeletal muscle, cardiac muscle
5	•		Skeletal muscle, brain tissue
6	•		Skeletal muscle
7	•		Skeletal muscle, brain tissue
8	•		Skeletal muscle
9	•		Skeletal muscle
11	•		Skeletal muscle
12	•		Skeletal muscle, cardiac muscle
13	•		None
15	•		Skeletal muscle
18	•		Skeletal muscle
19	•		None
21	•		Heart muscle
22		•	Skeletal muscle, cardiac muscle
23		•	Skeletal muscle, eye bulb
25		•	Skeletal muscle
30	•		None

Referring to the assessed hematological and hematochemical parameters, all sampled animals showed variations within physiological ranges and no statistically significant differences were highlighted between infected and uninfected ones. For this reason, values calculated for each parameter (Mean ± SD) are listed in Table-4 without distinguishing those obtained for infected animals from those obtained for uninfected ones.

**Table 4:** Hematological and biochemical parameters measured.

Hematological/biochemical parameters (measurement units)	Mean±SD
Hct (%)	46,4±9,1
Hgb (g/dL)	13,1±2,4
RBC (10^6^/μL)	7,0±1,5
WBC (10^3^/μL)	18,5±4,8
Neutrophils (%)	38,7±8,0
Lymphocytes (%)	67,8±14,7
Plt (10^3^/μL)	220±33
Glucose (mg/dL)	74,60±21,56
Total cholesterol (mg/dL)	88,6±18,91
Triglycerides (mg/dL)	58,0±16,02

Referring to the determination of serum 17-β estradiol (E2) concentrations, infected animals showed higher concentrations (p < 0.5) compared to uninfected ones. Specifically, 12.7 ± 0.14 pg/mL E2 and 11.7 ± 0.55 pg/mL E2 (Mean ± SD) were measured in animals which were positive to both diagnostic tests and in animals which were positive for one of them, respectively. In contrast, 8.6 ± 0.81 pg/mL were measured among negative animals.

Transcriptional responses detected for each factor were in some cases overlapping among different tissues while in other cases, opposite trends were highlighted. Among analyzed tissues, the brain was the one showing the greatest number of genes with a transcriptional change between infected and uninfected animals. Results are outlined below.

**Figure-1:**
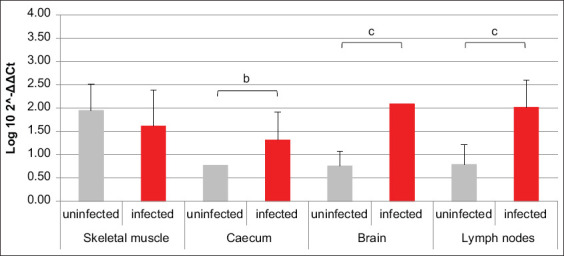
Bar plot representation of hypoxia-inducible factor 1α relative expression levels across pig tissues. Comparison between infected and uninfected animals. ^b^p < 0.01, ^c^p < 0.001.

**Figure-2:**
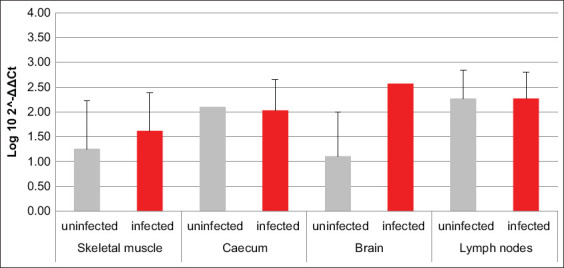
Bar plot representation of prolyl hydroxylases 2 relative expression levels across pig tissues. Comparison between infected and uninfected animals. ^c^p < 0.001.

HIF1α transcription was increased in brain, cecum, and lymph nodes of infected animals compared to uninfected ones, while an opposite trend was revealed in skeletal muscle (Figure-1). The amounts of mRNA coding for PHD2 were comparable between infected and uninfected animals, except for the brain, where they were higher in the first ones compared to the latter (Figure-2). Comparing the mRNAs coding for HIF1α and its inhibitor PHD2, differences among all four analyzed tissues were highlighted in terms of HIF1α/PHD2 ratio. In fact, in muscle of uninfected swine, HIF1α and PHD2 transcription levels were almost alike, while in infected animals, the second factor was greater than the first one. In cecum, brain tissue, and lymph nodes of uninfected swine, PHD2 transcription was increased compared to HIF1α. However, while in cecum of infected animals, their values became comparable, in the brain tissue of infected animals, their proportion was maintained due to an increase of both mRNAs. Finally, in lymph nodes of infected animals, the proportion between the two aforementioned mRNAs was reversed compared to uninfected animals, due to such an increase of HIF1α transcription levels as to exceed PHD2.

**Figure-3:**
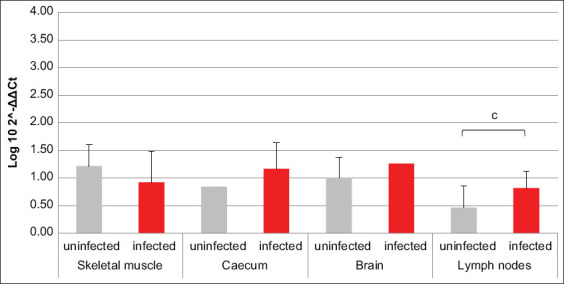
Bar plot representation of phosphatidylinositol 3-kinase relative expression levels across pig tissues. Comparison between infected and uninfected animals. ^a^p < 0.05, ^c^p < 0.001.

**Figure-4:**
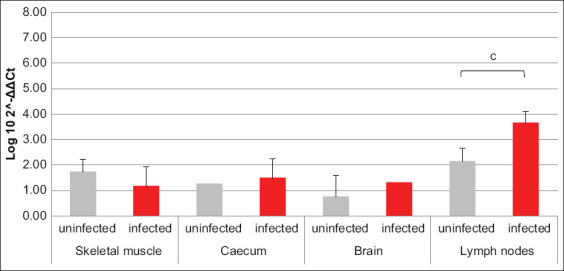
Bar plot representation of mitogen-activated protein kinase 3 relative expression levels across pig tissues. Comparison between infected and uninfected animals. ^c^p < 0.001.

**Figure-5:**
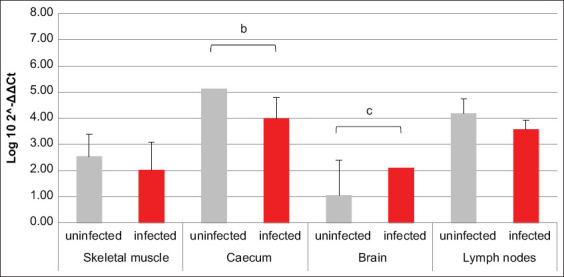
Bar plot representation of guanylate-binding protein 1 relative expression levels across pig tissues. Comparison between infected and uninfected animals. ^b^p < 0.01, ^c^p < 0.001.

**Figure-6:**
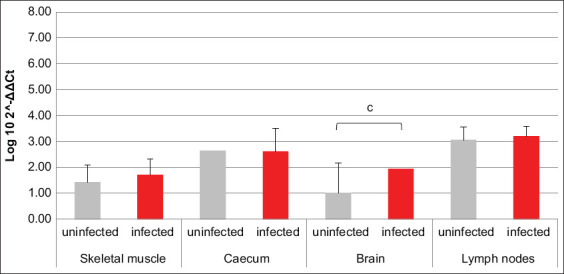
Bar plot representation of GBP5 relative expression levels across pig tissues. Comparison between infected and uninfected animals. ^c^p < 0.001.

**Figure-7:**
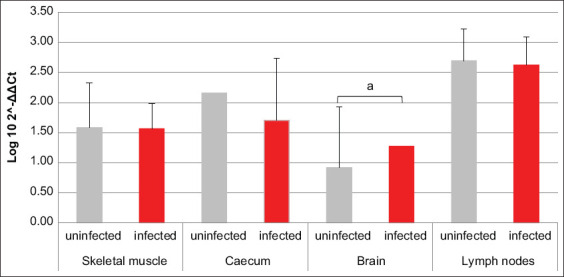
Bar plot representation of very large inducible GTPase relative expression levels across pig tissues. Comparison between infected and uninfected animals. ^a^p < 0.05.

**Figure-8:**
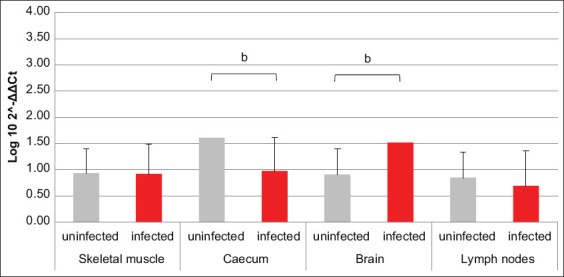
Bar plot representation of immunity-related GTPase C relative expression levels across pig tissues. Comparison between infected and uninfected animals. ^b^p < 0.01.

Referring to PI3K and MEK3, resembling results were obtained; in fact, for both factors, the mRNA transcription was increased in brain and lymph nodes of infected animals compared to uninfected ones, with an opposite trend revealed in skeletal muscle. Solely in cecum, the two factors did not return overlapping data. In fact, PI3K transcription was greater in infected pigs compared to uninfected, while MEK3 was almost unchanged between the two groups (Figures-3 and 4). Data about AKT1-coding mRNA were significant only when referring to muscle and cecum, where variations opposite to PI3K and MEK3 were revealed. The amount of NOD2-coding mRNA was smaller in cecum of infected swine compared to uninfected ones. Referring to GBP1, GBP5, VLIG, and IRGC, all encoding mRNAs were, respectively, almost unchanged in muscle and increased in brain of infected swine compared to uninfected animals. Moreover, GBP1- and IRGC-coding mRNAs were decreased in cecum of infected animals compared to uninfected ones (Figures-5–8).

## Discussion

*T. gondii* is a foodborne pathogen and infection is initially established in intestine following consumption of contaminated prey/food or water [16]. Among infected swine subjected to analyses, no parasitic DNA was detected in intestine, while its presence in other tissues was ascertained for most of them. Hence, considering the life cycle of *T. gondii*, it might be reasonably stated that infected swine sampled was at an advanced stage of infection at the time of slaughter and muscular contamination might be assumed as traceable to encysted forms of the parasite. Apart from three infected swine which tested positive solely to serological test and one in which *T. gondii* was detected exclusively in the heart, for most of the 19 infected pigs, the parasitic DNA was detected in skeletal muscle, and only in two cases, it was also detected in the brain, together with skeletal muscle. For this reason, while the transcriptional variations observed for skeletal muscle are certainly associated with the presence of *T. gondii*, in the other analyzed body districts, the transcriptional profile can be referred to as a state of infection but not necessarily as the parasitic contamination of the examined tissue. Based on the recorded evidences, various hypotheses can be formulated about the molecular mechanisms activated in swine at an advanced stage of infection by *T. gondii*. Referring to HIF1α and its inhibitor PHD2, it is well known that expression of the first factor is activated by the parasite in the early stages of infection and it plays a fundamental role in favoring *T. gondii* multiplication [12]. In this case, an increase in PHD2 and subsequently its inhibitory effect on HIF1α was observed. Considering that at the encysted stage, parasitic multiplication is reduced, data observed in skeletal muscles of infected swine suggest that at the encysted stage, when a new balance among host and *T. gondii* is established, the HIF/PHD2 physiological balance is restored. Similar data to those observed in skeletal muscle were also recorded for cecum and brain, while an overturning balance was recorded in the lymph nodes, with such an increased transcription of HIF1α as to exceeding PHD2. Considering that B cells are activated and differentiate under hypoxic conditions within lymph node germinal centers [17, 18] and that blood count did not veal an increase of circulating lymphocytes among infected swine compared to uninfected ones, data obtained should refer to HIF-elicited cascade activating the differentiation of memory B cells in lymph nodes of infected swine subjected to sampling.

Molecular factors involved in *T. gondii* recognition and in subsequent activation of downstream immune responses (NOD2 as a key receptor for inflammatory and immune responses, GBPs, IRGC, and VLIG as a key receptor for apoptosis and programmed cell death responses at the early stages of infection) were found unchanged between infected and uninfected animals in muscle. Considering what was previously mentioned regarding the state of infection of sampled pigs, evidences reported about the aforementioned receptors trace a metabolic framework favoring the maintenance of cysts, characterized by the downregulation of apoptosis in the host, which, in turn, favors a consequent limitation of the parasite replication.

A study published in 2018 [19] highlighted some key elements of the transcriptional responses to *T. gondii* during the acute phase of infection in cats. In particular, in various analyzed tissues (liver, lungs, small intestine, heart, brain, and spleen), a variation of the transcriptional profiles was highlighted for molecular factors such as PI3K/AKT1, NOD-like receptors, MAPK (mitogen-activated protein kinase) signaling pathway molecules, and GBPs. In addition, some authors also suggested that the transcriptional profiles may vary significantly even between the acute and chronic phases of infection. Hence, the aforementioned research helped to delineate a tissue specificity in response to infection and, on the other hand, deserves further investigation to be carried out also in other animal species. In fact, the transcriptional response can vary not only from tissue to tissue in the same species but also between different species, especially considering that *T. gondii*, albeit capable of infecting a wide range of host species, behaves differently in the definitive host (cat) compared to intermediate hosts. Unfortunately, the majority of studies performed on intermediate hosts refer to the most common animal models and usually come from observations performed during the acute phase of infection. Hence, due to the unavailability of the previous studies carried out on swine naturally infected, these results could not be compared to analogous data.

The analysis of hematological parameters did not reveal significant variations between infected and uninfected swine. To the best of our knowledge, rather scarce data are available about hematological and hematochemical evaluations in animals following *T. gondii* infection, except for similar assessments performed on sheep [20]. This study revealed comparable results referring to hematological parameters and a significant discrepancy related to the blood glucose determination. In fact, a significant decrease in blood glucose was found in infected sheep compared to uninfected ones [20], while no similar data were observed in swine. According to references, host glucose is an essential substrate for *T. gondii* multiplication, exploited as the first and most efficient energy source [21, 22]. Unfortunately, no sufficient data and experimental results are available about the exploitation of this substrate in other host species; hence suggesting an objective scientific interpretation in this regard is quite difficult. A significant variation of E2 concentrations was revealed between infected and uninfected swine so, referring to *T. gondi*i infection in swine, our results confirm the involvement of sex hormones in host response to infections suggested in references [9] .

## Conclusion

Gene expression outlined by means of RT-PCR assays revealed differences between infected and uninfected animals and, referring to the formers, differences between the various analyzed tissues were also highlighted. Data reported in this study constitute a contribution to deepening the cellular and molecular pathways activated in response to toxoplasmosis in swine. Such evaluations need to be further investigated not only referring to pigs but also to other species, such as sheep among which *T. gondii* can exert a relevant clinical action with a considerable economic impact for breeders. Furthermore, additional data could lead to comparisons between different species and help to understand which elements determine a greater resistance in some species such as equines. This study, in addition to providing useful data for a better understanding of the host-parasite interaction, may assist the selection of biomarkers useful for developing new diagnostic tools or formulating therapeutic aids to eradicate this zoonosis.

## Authors’ Contributions

AMFM: Conceived the idea of the study. AMFM, EF, and AMF: Designed the study. RPG and AS: Sample collection. AC and TA: Performed the biomolecular assays and processed raw data for the evaluation of expression profiles. RPG: Serological diagnosis of toxoplasmosis. CC and PM: Performed the 17-β estradiol dosage and analyzed raw data. GB: Assessed the hematological and hematochemical parameters and analyzed raw data, AMF: Assessment of hematological and hematochemical parameters. AMFM: Oversaw all stages of the present study. AC: Drafted the manuscript. AMFM and AC: Revised this manuscript. All authors have read and approved the final manuscript.
